# *Mycoplasma pneumoniae*: current outbreak

**DOI:** 10.1017/S0950268824000293

**Published:** 2024-02-21

**Authors:** Tim Wreghitt

**Keywords:** Mycoplasma pneumoniae, outbreak, epidemic

*Mycoplasma pneumoniae* causes outbreaks of respiratory infection throughout much of the world every 3–5 years. It causes a range of symptoms of which a prolonged dry cough is the most prominent. Unlike winter viral respiratory infections, *M. pneumoniae* has an incubation period of 1–4 weeks, which results in slow lumbering outbreaks. It is important to identify the beginning of a new outbreak as soon as possible in order to review antibiotic prescribing policies (especially in the community) and to ensure that appropriate laboratory diagnostic methods are in place on appropriate specimens.

Accompanying this Editorial is a report of a hospital outbreak in France (Zayet et al.) from November 2023 to January 2024 which provides a valuable wake-up call. There have been other reports of infections across Europe and Asia and surveillance data suggest that we are in an epidemic winter for *M. pneumoniae* infection this year. Clinicians have been advised in the United Kingdom to follow the current National Institute for Health and Care Excellence guidance for the treatment of pneumonia but there must be an awareness of the antibiotic sensitivities of *M. pneumoniae*, which does not respond to penicillins. This is particularly important in the community.

Another important aspect is laboratory diagnosis. The most rapid means of accurate diagnosis is by use of molecular methods, which can provide a rapid and sensitive result if the correct specimens are submitted. However, it is important that the laboratory methods employed are available to doctors in the community (especially General Practitioners) and that these medical staff are aware of the importance of rapid diagnosis, which enables appropriate antibiotic prescribing, and are aware of which specimens to submit.

If molecular diagnostic tests are unavailable locally or are negative in patients with suspected *M. pneumoniae* infection, another means of laboratory diagnosis is by looking for *M. pneumoniae* antibodies. Since *M. pneumoniae* outbreaks occur every 3–5 years and immunity is not lifelong, we are all likely to experience infection on several occasions during our lifetimes, typically as a schoolchild, then as a parent and finally, as a grandparent. If *M. pneumoniae* IgM antibody detection methods are employed, it is important to remember that in 100% of people experiencing their first *M. pneumoniae* infection, specific IgM will be detectable. However, those with a second infection, years later, may not produce any specific IgM or produce only small amounts and it is unlikely that those older people experiencing further infection will produce specific IgM. It is for this reason that *M. pneumoniae* IgM tests must be interpreted with extreme caution, especially in adults and elderly persons.

It is most unusual to have reports of localized outbreaks of *M. pneumoniae* as in the accompanying paper by Zayet et al. Furthermore, it seems to be behaving unlike the usual lumbering pattern of a slow rise and fall over about every 4 years.

We endorse Zayet’s and his colleagues’ recommendation that worldwide surveillance for *M. pneumoniae* is needed at the present time. Further reports of other *M. pneumoniae* and other respiratory agents to *Epidemiology and Infection* are welcome.

